# The Serious Impact of Comorbidities on Methemoglobinemia: A Case Report

**DOI:** 10.7759/cureus.33923

**Published:** 2023-01-18

**Authors:** Antonio Eliseu, Carolina Palma, Margarida Agudo, Miguel Rodrigues, Francisca Santos

**Affiliations:** 1 Internal Medicine, Centro Hospitalar de Setúbal, E.P.E., Setúbal, PRT

**Keywords:** comorbidities, altered mental status, respiratory failure, status epilecticus, methemoglobinemia

## Abstract

Methemoglobinemia is a rare, life-threatening condition that occurs when the body is exposed to oxidative stress. We present the case of a 72-year-old female with a past medical history of hypertension, obesity, dyslipidemia, and heart failure who was admitted to the emergency department with altered mental status and respiratory failure. After admission, we also identified an atrioventricular block 2:1, anemia, and skin discoloration. We performed endotracheal intubation and started mechanical ventilation due to respiratory failure; however, the patient retained an oxygen "saturation gap" despite adequate ventilation. In the initial laboratory evaluation, methemoglobinemia was found to be 13%, reaching a maximum level of 16%. An electroencephalogram revealed status epilepticus after her admission to the intensive care unit. Despite all efforts and supportive care, methylene blue therapy was never attempted, and the patient died. Our case emphasizes the importance of a high index of suspicion for methemoglobinemia, especially in the presence of an oxygen "saturation gap," and that despite relatively low levels of methemoglobinemia, it can have a more severe clinical presentation in patients with comorbidities. In these patients, a reduced threshold for administering methylene blue should be taken into account.

## Introduction

A biologically formed type of hemoglobin known as methemoglobinemia (MetHb) is created when the heme iron is oxidized from the ferrous (Fe2+) state to the ferric (Fe3+) state. Hypoxemia results from this Fe3+ state's poor ability to bind oxygen [1]. If untreated, MetHb is a disorder that can be serious or even fatal [2]. MetHb is classified into two types: hereditary and acquired [2,3]. Anemia, congestive heart failure, chronic obstructive pulmonary disease, and essentially any pathology that impairs the ability to deliver oxygen may worsen the symptoms of MetHb [4].

The percentage of methemoglobin is calculated by dividing methemoglobin concentration by total hemoglobin concentration. Because underlying medical conditions play an important role (e.g. anemia), the percentage of methemoglobin is likely a better indicator of illness severity than overall concentration.

We present a rare case of methemoglobinemia with a more severe clinical presentation than expected. Despite reaching a maximum of 16% MetHb, her comorbidities most likely exacerbated the clinical picture.

## Case presentation

We present the case of a 72-year-old Caucasian female with a medical history of hypertension, obesity, dyslipidemia, and heart failure who was chronically treated with ramipril/amlodipine 5/10 mg qd, rosuvastatin 10 mg qd, and furosemide 40 mg qd.

She was admitted to the emergency department due to an altered mental status. She was found unconscious on her property by an emergency medical vehicle team after neighbors spotted her lying in the garden. The duration of this state could not be determined. The family could not be contacted upon the arrival of the emergency team or in the emergency room (ER). Therefore, the only medical history available was obtained through access to previous medical records.

She was admitted with an oropharyngeal airway device and high-flow oxygen mask due to low oxygen saturation (SaO_2_). She had altered consciousness (Glasgow Coma Scale = 3), peripheral arterial oxygen saturation measured by pulse oximetry (SpO_2_) of 86% (high-flow oxygen mask, 15 L/min), and blood pressure of 80/40 mmHg. An electrocardiogram revealed a heart rate of 40 beats per minute (bpm) with an atrioventricular (AV) block of 2:1. The physical examination showed a comatose patient with non-photoreactive and slightly mydriatic pupils, no corneal reflex, and generalized symmetrical hypotonia. It should also be noted that after two hours, normal skin color became cyanosed in the extremities.

The arterial blood gases (ABGs) showed severe uncompensated respiratory acidemia (pH 7.15, partial pressure of carbon dioxide (PaCO_2_): 84 mmHg), elevated partial pressure of oxygen (PaO_2_: 270 mmHg), hyperkalemia (5.9 mmol/L), slightly elevated bicarbonate (HCO_3_: 29.3 mmol/L), and SaO_2_ of 94% (Table 1). It is important to note that the blood specimen for ABG had a "dark brown" color and the pulse oximetry measurement had a good perfusion index and curve, with SpO_2_ always under 90% (84-89%), despite the very high levels of PaO_2_ in ABG, SaO_2_ of 94%, normal lactate (1.2 mmol/L), and a methemoglobin level of 13%. Her blood tests revealed that she had a slight leukocytosis (13 µL) and anemia (10.1 g/L) (Table 2). 

**Table 1:** Arterial blood gases. ABG 1: arterial blood gas 1-FiO_2_ 100% via high-flow oxygen non-rebreather mask; ABG 2: arterial blood gas 2-FiO_2_ 100% via mechanical ventilation; PaO_2_: partial pressure of oxygen; PaCO_2_: partial pressure of carbon dioxide; HCO_3_: bicarbonate; SaO_2_: oxygen saturation; FiO_2_: fraction of inspired oxygen.

Variable	Reference range	Results (ABG 1)	Results (ABG 2)
pH	7.35–7.45	7.15	7.47
PaCO_2 _(mmHg)	35-45	84	42
HCO_3 _(mmol/L)	22.0–28.0	29.3	22.6
PaO_2 _(mmHg)	75–100	270	132
SaO_2 _(%)	95–100	94	94
Glucose (mg/dL)	70–110	156	126
Sodium (mmol/L)	136–145	137	138
Potassium (mmo/L)	3.50–5.10	5.9	3.9
Chloride (mmol/L)	98–107	103	103
Ionized calcium (mmol/L)	1.12–1.30	1.18	1.19
Lactate (mmol/L)	0.2–2.0	1.2	2.1
Methemoglobin saturation	<1.5%	13	16

**Table 2:** Initial investigation. ALP: alkaline phosphatase; ALT: alanine aminotransferase; AST: aspartate aminotransferase; CRP: C-reactive protein; GGT: gamma-glutamyl transferase; LDH: lactic dehydrogenase; MCH: mean corpuscular hemoglobin; MCV: mean corpuscular volume.

Variable	Reference range	Results
Hemoglobin (g/dL)	12.0–15.0	10.1
MCV (fL)	78.0–96.0	83.0
MCH (pg)	26.0–33.0	28.1
White cells count (per µL)	4,500–11,000	13.1
Neutrophils (per µL)	2,000–8,500	9,760
Lymphocytes (per µL)	900–3,500	2,130
Platelets (per µL)	1,50,000–4,50,000	2,30,000
CRP (mg/dL)	<3.0	2.3
Urea (mg/dL)	15.0–40.0	39
Creatinine (mg/dL)	0.57–1.11	0.9
Sodium (mEq/L)	136–145	137
Potassium (mEq/L)	3.50–5.10	4.2
Chloride (mEq/L)	98–107	103
AST (U/L)	5.0–34.0	39
ALT (U/L)	0.0–55.0	37
GGT (U/L)	9.0–36.0	21
ALP (U/L)	40–150	52
LDH (U/L)	125–220	170
Bilirubin (mg/dL)	0.20–1.20	0.8

We administered insulin, glucose, and calcium gluconate for hyperkalemia and atropine and isoprenaline for bradycardia. Following these interventions, the AV block was converted to sinus bradycardia at 50 bpm, and the hypotension was reversed (127/73 mmHg). The patient was intubated due to respiratory failure and for airway protection. Midazolam and rocuronium were administered for rapid sequence intubation, and the patient was placed on the volume control mode of ventilation (VC: 480 mL, positive end-expiratory pressure (PEEP): 8, respiratory rate: 15 bpm, fraction of inspired oxygen (FiO_2_): 100%). Blood gas analysis was repeated, revealing reversal of acidemia (pH: 7.47), normocapnia (PaCO_2_: 42 mmHg), and elevation of PaO_2_ (132 mmHg). A nasogastric tube was placed, and 300 mL of clear "gasoline" smell content was drained, which could have been the source of MetHb; however, a positive result from the toxicology laboratory was never obtained.

A contrast-enhanced head CT revealed no acute intra- or extra-axial changes, and the major supra-aortic arterial vessels were permeable and thrombus-free. The chest X-ray was normal. The patient was admitted to the intensive care unit, where she reached a maximum methemoglobin level of 16%. An electroencephalogram was performed due to the patient's unresponsiveness and normal head imaging studies, which revealed non-convulsive status epilepticus (NCSE).

She was started on antiepileptic drugs and progressed through up to five different classes at maximally tolerable doses without a clinical or electrographic response. Methylene blue administration was never attempted. The serious clinical state caused by MetHb and aggravated by her comorbidities led to the need for mechanical ventilation that was maintained for a week before ventilator-associated pneumonia developed, which ultimately precipitated the patient's death. 

## Discussion

Methemoglobin develops when iron in hemoglobin is oxidized from the ferrous (Fe2+) state to the ferric (Fe3+) state. When the iron of hemoglobin is oxidized to Fe3+, it is unable to carry oxygen. Increased levels of methemoglobin result in functional anemia [1]. If untreated, MetHb is a disorder that can be serious or even fatal [2]. MetHb is classified into two types: hereditary and acquired [2,3]. Exposure to chemicals that either directly or indirectly oxidize hemoglobin results in acquired MetHb, which is by far the most prevalent type. Because of this exposure, more methemoglobin is produced than the body can use to revert the iron in hemoglobin to its Fe2+ state [4].

Unfortunately, in our case, we could not identify the suspected ingested substance that led to MetHb. The patient was found in her garden, which may suggest she was eventually exposed to an unidentified fertilizer or dye. Other components of the unknown toxicant substance consumed could cause direct toxicity, further exacerbating the clinical picture. 

Methemoglobin is represented as a concentration or a percentage. By dividing the concentration of methemoglobin by the concentration of total hemoglobin, the percentage of methemoglobin is obtained. The percentage of methemoglobin is probably a better predictor of illness severity than overall concentration. This may be exacerbated by the decreased ability of "functional hemoglobin" to release oxygen in the presence of methemoglobin.

Certain patient populations are more vulnerable to MetHb and should be closely monitored and more aggressively treated when MetHb is suspected. Comorbidities that impair oxygen transport, such as anemia, heart disease, and pulmonary disease, predispose and worsen patients' clinical status [5,6].

Our patient did have anemia and heart failure, which put her at a higher risk of developing more severe symptoms of MetHb. 

A clinical diagnosis of MetHb is made based on the patient's medical history and current symptoms, such as hypoxia that is resistant to oxygen supplementation and possibly chocolate-colored blood. An arterial or venous blood gas test with co-oximetry is used to confirm the diagnosis. This test differentiates hemoglobin by determining the concentration and percentage of methemoglobin [7]. It is impossible to determine the severity of MetHb with SpO_2_ readings. The term "refractory hypoxemia" is a crucial diagnostic indicator. This is typically visible on SpO_2_ (pulse oximetry measurements) based on wavelength detection, but not on calculations utilizing PaO_2_ and SaO_2_ (on blood gas analyses). Whereas SaO_2_ estimates are incorrectly normal because it is assumed that all hemoglobin is either oxyhemoglobin or deoxyhemoglobin, SpO2 measurements are low and depressed by wavelength interference, frequently to 75-90% even with more oxygen [7]. The "saturation gap" refers to the discrepancy between the low SpO_2_ reading and the erroneously normal SaO_2_ estimate.

 In our case, the patient had "chocolate-colored blood," as well as SpO_2_ measurements of 84-88% and an ABG with a SaO_2_ of 94%, which is a very important diagnostic clue for MetHb, confirming the oxygen "saturation gap."

Cyanosis can be clinically evident in otherwise healthy people with methemoglobin levels as low as 10% [6]. The classic appearance of "chocolate brown blood" can be found in as little as 15%. As the methemoglobin level approaches 20%, the patient may experience anxiety, lightheadedness, and headaches. At 30-50%, tachypnea, confusion, and loss of consciousness may occur. There is an increased risk of seizures, dysrhythmias, metabolic acidosis, and coma when the level approaches 50%. Despite this, in patients with comorbidities, previous reports suggested that even levels below 20% could lead to status epilepticus [8]. Levels above 70% are frequently fatal [6,9]. 

This case report highlights the importance of comorbidities and their impact on disease severity. Our patient only reached a maximum level of 16% of MetHb; however, she presented with a very severe clinical presentation: NCSE, AV block, and acute respiratory failure. She had anemia (10 g/L) and a past medical history of heart failure, which, as the literature shows, probably exacerbated the symptoms [6,7,10]. Regarding NCSE, it could be related to MetHb neurotoxicity in combination with hypoxemia and other toxicant components of the unidentified substance as a plausible cause. In otherwise healthy individuals, this kind of severe presentation is usually seen when MetHb is around 50%.

To treat MetHb, the provoking factor must be eliminated, and the antidote methylene blue (MB) may also be used (tetramethylthionine chloride). By using a non-rebreather mask to give high-flow oxygen, tissues receive more oxygen, and methemoglobin is naturally degraded more quickly. MB often operates quickly and efficiently through its involvement with the secondary pathway of methemoglobin reduction. When methemoglobin surpasses 20-30% in cases of acquired MetHb, or at lower levels if the patient is symptomatic, MB treatment should be administered. The dosage of MB is 1-2 mg/kg intravenously over a five-minute period (0.1-0.2 mL/kg of a 1% solution) [10]. Other treatment options are ascorbic acid, transfusion, exchange transfusion, or hyperbaric oxygen. Ascorbic acid has a reducing potential and may be useful in situations where MB cannot be used, such as glucose-6-phosphate dehydrogenase (G6PD) deficiency or in a person receiving a serotonergic agent. If the patient does not respond to MB or ascorbic acid, transfusions, exchange transfusion, and hyperbaric oxygen may be tried [11-13].

In our case, MB was not administered, which in retrospect should have been provided since the patient was symptomatic despite relatively low levels of MetHb. The serious clinical state caused by MetHb, exacerbated by her comorbidities, necessitated mechanical ventilation for a week before ventilator-associated pneumonia developed, resulting in the patient's death. 

## Conclusions

MetHb is a rare and potentially fatal cause of death if not addressed quickly or left untreated. Our case shows the importance of the "saturation gap" and chocolate-colored blood as an important diagnostic hint. Also, this case emphasizes the importance of the patient's previous comorbidities in determining the severity of the disease. In these patients, treatment choices should not be deferred based on confirmatory laboratory results but rather on clinical presentation, and a lower threshold for the administration of MB should be considered. Identification of the toxicant is also critical in order to manage our patients as effectively as possible, which was unfortunately not possible in our case.
